# Comparison of early and delayed invasive strategies in short-medium term among patients with non-ST segment elevation acute coronary syndrome: A systematic review and meta-analysis

**DOI:** 10.1371/journal.pone.0220847

**Published:** 2019-08-12

**Authors:** Ming-Bo Zhang, Chen Guo, Min Li, Yong-Hui Lv, Yu-Dong Fan, Zhi-Lu Wang

**Affiliations:** 1 The First Medical Clinical College of Lanzhou University, Lanzhou, China; 2 Department of Cardiology, Emergency General Hospital, Beijing, China; 3 Department of Cardiology, The First Hospital of Lanzhou University, Lanzhou, China; Hospital Clinico San Carlos, SPAIN

## Abstract

**Background and objectives:**

An invasive approach is recommended as the treatment of patients with non-ST elevated acute coronary syndromes (NSTE-ACS). However, it remains unclear that the optimal time of angiography and intervention for patients with NSTE-ACS at present. This study was designed to compare the effect of early and delayed invasive strategies on short-medium term prognosis in patients with those.

**Methods:**

Pubmed, Cochrane Library and Embase were searched up to Dec-30-2018. Randomized clinical trials comparing an early versus a delayed invasive strategy in patients with NSTE-ACS were included. The primary endpoint (all-cause death and recurrent myocardial infarction) and secondary endpoint (major bleeding and recurrent revascularization), as well as composite endpoint were assessed by random or fixed effected meta-analysis with software RevMan 5.3 version after short-medium term follow up.

**Result:**

A total of six randomized clinical trials involving 4,277 early or delayed invasive strategies patients with NSTE-ACS were included in the meta-analysis. Time to coronary angiography varied from 0.5 to 24 h in the early invasive strategy and from 18.6 to 72 h in the delayed invasive strategy. There was a statistical difference in the primary endpoint of all-cause death among patients with NSTE-ACS between early and delayed invasive strategies (4.6% vs 6%; OR:0.76; 95% CI:0.58 to 1.00; *P* = 0.05; *I*^*2*^ = 0%), but not for recurrent myocardial infarction (6.0% vs 6.3%; OR: 0.94; 95% CI: 0.55 to 1.61; *P* = 0.82; *I*^*2*^ = 60%). The major bleeding in patients with NSTE-ACS was similar between both invasive strategies (2.7% vs 3.1%; OR:0.88; 95% CI:0.59 to 1.31; *P* = 0.54; *I*^2^ = 0%). However, the composite endpoint in the early invasive strategy patients with NSTE-ACS was significantly lower than that of the delayed invasive strategy (10.9% vs 13.9%; OR:0.76; 95% CI:0.63 to 0.92; *P* = 0.006; *I*^2^ = 0%), and the recurrent revascularization between both strategies was just the opposite (8.7% vs 5.9%; OR:1.5; 95%CI:1.15 to 1.97; *P* = 0.003; *I*^*2*^ = 0%).

**Conclusion:**

The systematic review and meta-analysis demonstrated that the early invasive strategy had a beneficial trend on all-cause death and significantly reduced the composite endpoint in patients with NSTE-ACS, but increased the rate of revascularization. These data could provide a solution for patients with those.

## Introduction

Acute coronary syndrome (ACS) is mainly caused by atherosclerotic plaque rupture or intraluminal thrombus in one or more coronary arteries decreasing the infusion flow and leading to myocardial necrosis [1]. Non-ST segment elevation myocardial infarction (NSTEMI) and unstable angina were included in the non-ST elevated acute coronary syndromes (NSTE-ACS). The incidence of NSTEMI has slightly increased compared with ST segment elevation myocardial infarction (STEMI) over the last decade due to new risk factors and demographic changes [2]. The mortality caused by NSTE-ACS was twice as much as that of ST-segment elevation myocardial infarction [3]. The quality of life and mental health for patients with NSTE-ACS were seriously affected by mortality and heart-related complications.

An invasive approach was superior to a conservative or a selective invasive approach in preventing death and myocardial infarction among patient with NSTE-ACS [4]. Timely intervention therapy is essential to improve prognosis of patients with NSTE-ACS. However, the optimal timing of routine intervention is still unclear in long-term follow up. Previous meta-analysis indicated that the incidence of recurrent myocardial ischemia could be reduced and shorten days of hospitalization patients with NSTE-ACS by early intervention with 1 month follow up, but there was no significant difference in death and re-infarction rate in patients with those between early and delayed invasive strategies [5,6]. The delayed invasive strategy showed that the unstable coronary plaques could be passivated by the optimal drugs during the waiting period of delayed invasive treatment, which could prevent the recurrence of myocardial ischemia caused by plaque shedding during the intervention [7]. Therefore, it was not advocated to intervene prematurely or to delay too much [8]. In addition, the invasive intervention time recommended by the current guideline was mainly based on expert opinion or primarily a pre-specified subgroup analysis [5].

This study was designed to obtain optimal time for routine invasive treatment and long-term evidence to all-cause death by comparing adverse cardiovascular events in patients with NSTE-ACS with early and delayed invasive treatment after short-medium term follow-up. Simultaneously, this study hypothesized that early invasive strategy could benefit from all-cause death and other clinical endpoints and will provide evidences for the development of the guideline.

## Methods

### Data source and search strategy

PubMed, Cochrane Library and Embase database were searched from building to Dec-30-2018. All retrieval was not restricted by language and date of publication. Search strategies included Medical Subject Heading (MeSH) terms and keywords. The MeSH terms were non-ST-elevated myocardial infarction and percutaneous coronary intervention, randomized controlled trial. The keywords included acute coronary syndrome, ACS, non-ST-elevation acute coronary syndromes, NSTE-ACS, non-ST-elevated myocardial infarction, NSTEMI, percutaneous coronary intervention (PCI), PCI, percutaneous coronary revascularization, early or delayed intervention, invasive intervention, coronary invasive strategy, randomized controlled trials (RCTs) and RCTs. Clinical trial registries (ClinicalTrials.gov and www.controlled-trials.com) and the web for relevant abstract or presentation from major cardiovascular meetings was researched for published and unpublished studies. Additionally, the references of relevant articles and reviews were also scanned. The combined mode of MeSH or keywords was also retrieved. The titles and abstracts of the relevant publications were manually reviewed to exclude unrelated and duplicated articles (Zhang MB, Guo C, Lv YH and Li M). The report of the methods in this article was in accord with this principle that the Preferred Reporting Items for Systematic Reviews and Meta-Analyses (PRISMA) consensus statement for the randomized controlled trials [9]. All analyses were based on previous published some studies, thus no ethical approval and patient consent are required.

### Study eligibility

Patient and outcome data were retrieved by two independent investigators (Li M, Lv YH). Unrelated articles were excluded by reading titles and abstracts of literatures. If articles were relevant to the research topic, the full text was retrieved for reference. The full text was carefully read and determined that whether the article was included or excluded according to the inclusion and exclusion criterion by two investigators (Li M, Lv YH). In the process of full text filtering, if the relevant information was not complete, the corresponding author was contacted via email to acquire. Discrepancy in the inclusion were resolved by discussion (Li M, Lv YH, Guo C, and Zhang MB) or consulted with the third parties (Wang ZHL, and Fan YD). Studies were eligible for inclusions if they met all of the following criterion: (1) enrolled patients ≥18 years of age; (2) the included patients diagnosed with NSTE-ACS; (3) comparing early versus delayed invasive strategies; (4) a randomized control trial; (5) the definition of early and delayed time was based on the time of coronary angiography: the strategies included all early intervention and all delayed intervention in relevant trials; (6) the strategy of invasive treatment was defined as coronary angiography, and percutaneous coronary intervention or coronary artery bypass grafting (CABG) according to the results of coronary angiography; (7) the durations of follow-up was at least 3 months. Studies were excluded to reduce bias if they met one of the following criterion: (1) observational studies; (2) ongoing randomized controlled trials; (3) patients diagnosed with ST-elevation myocardial infarction; (4) comparing a routine invasive strategy versus conservative or selected strategy; (5) the duration of follow-up was less than 3 months; (6) no relevant information on this research was provided. The studies including the reported incidence of all-cause death, recurrent myocardial infarction, myocardial ischemia, major bleeding and recurrent revascularization were eligible for the meta-analysis.

### Clinical endpoints

Primary endpoints were composed of all-cause death and recurrent myocardial infarction. Secondary endpoints included a composite endpoint (all cause death, recurrent myocardial infarction, myocardial ischemia), major bleeding and recurrent revascularization. The definition of myocardial infarction was consistent with each original trial. The definitions of myocardial infarction from 6 randomized controlled trials were shown in Table 1. The major bleeding was defined as shock, the need of the transfusion of ≥2 unite red blood cells, intracranial hemorrhage, a fall of hemoglobin ≥5 g/dl, or vascular surgery for bleeding complications. The definitions of the endpoint for the trials were not described in detail for other studies [10,11].

**Table 1:** The definitions of myocardial infarction including recurrent MI in the meta-analysis.

ELISA3 2016	LIPSIA 2012	OPTIMA 2016	Sciahbasi 2010	Tekin 2013	TIMACS 2009
Early reinfarction With CK-MB > ULN: A decrease in CKMB of at least 50% of ULN from a prior peak level to a valley followed by a new increase with a value above the sum of the preceding valley and three times the ULN. The development of new Q-waves in> 2 contiguous leads. Early reinfarction with normal CKBM As a peak CKMB greater than three times the ULN with the exception of cases where the CKMB release curve was unequivocally related to the chest pain episode before randomization and not to the chest pain episodes after randomization or development of new Q-waves in>2 contiguous leads. Late reinfarction with CKMB returning to normal: As peak CKMB greater than three times the ULN or the development of new Q-waves in >2 contiguous leads. MI in patients who underwent CABG was defined as the new Q-waves in>2 contiguous leads.	Re-MI in hospital defined by the occurrence of any of the following: New Q waves in 2 contiguous leads plus ischemic symptoms >20 minutes New ST-segment elevation in 2 contiguous leads plus ischemic symptoms >20 minutes OR CK-MB >5 times ULN (An increase>50% was required if CKMB was >5 ULN at randomization) Post discharge re-MI: Ischemic symptoms and troponin > 99th percentile ULN	MI during hospitalization: any rise in CKMB >ULN. Outpatient follow-up: ≥2 of Chest pain >20 min. New pathological Q waves or New ST-segment elevation of ≥1 mm in two contiguous leads.	No definition given	Elevation of cardiac markers during the post hospitalization period, along with chest pain relevant to ischemia or ischemic ECG changes.	MI within 24 h: Symptoms >20 min and New ST-elevation or depression >0.1 mV in >2 contiguous leads MI within 24 h-7 days: Symptoms >20 min and CKMB >2 ULN or >50% above previous valley level with already elevated biomarkers or dynamic ST change in two or more contiguous leads. No biomarker elevation baseline: Any increase in biomarkers >2 ULN plus ≥1 of the following: Ischemic symptoms Development of pathological Q waves ECG changes indicative of ischemia Coronary intervention Pathological findings of an MI >7 days: similar to no biomarker elevation at baseline After PCI: CKMB >3 ULN or Increase >50% from pre-procedural valley level and >3 ULN with already elevated enzymes or New ST-elevation or development of significant Q waves in >2 contiguous leads.

**Abbreviations**: ULN: Upper limit of normal; CKMB: Creatine kinase myocardial isoenzyme.

### Data extraction and quality assessment

Data extraction and quality assessment were performed independently by two reviewers (Zhang MB, Guo C). A standard data extraction form was established and two reviewers were trained according to the rules of data extraction before data collection. Missing data were requested by the correspondence authors providing information on the published studies and trials. During the process of the data extraction, extractor was blind to the relevant information on included trials to reduce the bias. Disagreements were resolved by consensus or a third parties (Wang ZHL and Fan YD). Relevant information was extracted from each trial: the first author, study characteristics, the publication date of studies, the method of design, the duration of follow-up, baseline characteristics, procedural characteristics, medicinal therapy and clinic outcomes. The general quality of each selected randomized controlled trial was evaluated using the validated criteria proposed by Cochrane Collaboration’s tool [12].

### Statistical analysis

Data were analyzed by statistical software RevMan 5.3 version according to the intention-to-treat. The data about the outcome were dichotomous. Odds ratio (OR) and 95% confidence intervals (CI) were calculated. The OR was defined as the ratio of the odds of the studied event (all-cause death, recurrent myocardial infraction, composite endpoint, major bleeding, recurrent revascularization). Each outcome was analyzed with OR between the early and the delayed invasive strategies. A fixed or random effects model was applied to accommodate for heterogeneity between both strategies. The heterogeneity was assessed by the *I*^*2*^ statistic, which represented the percentage of variance due to the factors of the included studies rather than the sampling error. If *I*^*2*^ was more than 50%, which meant there was a large of heterogeneity and the random effects model (Mantel-Haenszel method) was performed. Otherwise, the fixed effected model was adopted. Funnel plots were used to estimate the risk of publication bias. Sensitivity analysis was performed to address the impact of each study on the combined effect, by testing whether removing one study at the same time would alter the results of the meta-analysis to find potential sources of heterogeneity. All *P* values were two sided, a *P* value less than or equal to 0.05 was considered statistically significance.

## Results

### Search results and study characteristics

**Fig 1:**
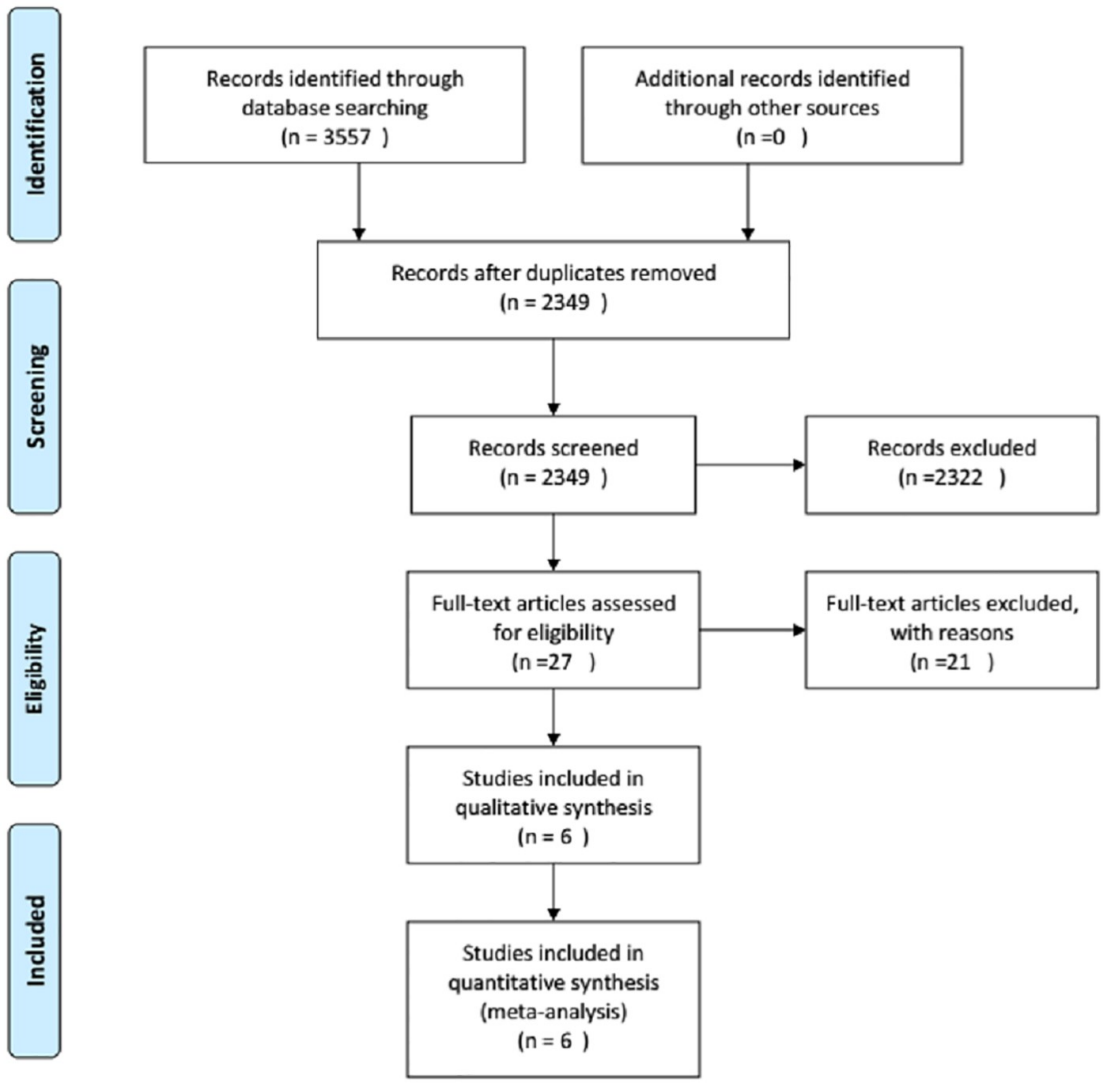
Summary of literature searching process.

The results of searching progress are illustrated (Fig 1). A total of 27 articles were included, 21 of which were removed by previewing full text with reason: editorials, literature reviews, observational studies, routine invasive versus conservative strategies, systemic reviews, ongoing trials. The final six randomized controlled trials contained 4,277 patients with NSTE-ACS were eligible for inclusion in the meta-analysis. Among them, the early invasive strategy was performed for 2,224 patients and the delayed invasive strategy was performed for 2,053 patients. The baseline characteristics of the patient included in the study are presented in Table 2. All patients in trials were assigned to the early and delayed invasive strategies, except for the LIPSIA trials [10]. Patients in the LIPSIA trial were assigned to either an immediate, early, or selective invasive strategy. The selective group to keep in line with the included criteria was excluded. Each trial was well-matched between both strategies.

**Table 2:** Baseline characteristic for patient included study.

Characteristic	ELISA3 2016		LIPSIA 2012		OPTIMA 2016		Sciahbasi 2010		Tekin 2013		TIMACS 2009	
	Early	Delayed	Early	Delayed	Early	Delayed	Early	Delayed	Early	Delayed	Early	Delayed
**Follow-up (m)**	24		6		60		12		3		6	
**TTA (h)**	2.6	54.9	1.1	18.3	0.5	25	5	24	<24	24–72	14	50
**Age (years)**	72.1	71.8	68	70	63	62	58.8	59.7	58.1	55.6	65	65.7
**Male (%)**	69.5	67.5	66	70	70	74	81.5	88.9	59.4	71.2	65.2	65.4
**Diabetes (%)**	23.8	20.4	39	43	19	20	26	18.5	31.9	45.2	26.5	27.4
**HPL (%)**	NP	NP	40	42	38	32	51.9	55.6	62.3	50.1	NP	NP
**HTN (%)**	54.3	58.1	82	82	53	33	48.2	66.7	55.1	50.2	NP	NP
**Smoking (%)**	21.2	26.4	29	25	38	39	59.3	44.4	60.9	48.4	NP	NP
**Pre MI (%)**	17.8	19.6	18	24	21	26	NP	NP	NP	NP	19.7	20.9
**PreStroke (%)**	3.3	4.5	5	6	NP	NP	NP	NP	NP	NP	7.2	7.5
**PreCABG (%)**	13.8	12.1	5	8	11	1	NP	NP	NP	NP	7	7.3
**Pre PCI (%)**	18.2	20.8	16	16	27	19	NP	NP	NP	NP	13.9	14.2

**Abbreviations**: TTA: time to angiography; HPL: Hyperlipemia; HTN: Hypertension; Pre: Perious; MI: myocardial infarction; CABG: coronary artery bypass grafting; PCI: percutaneous coronary intervention; NP: Not provided.

The average age of these included patient was 64.1±5.3 years in two invasive strategies. The included patients with diabetes, hypertension accounted for 27.7% and 18.4% respectively. The patients with diabetes had a similar proportion (28.2% vs 27.3%) between two invasive strategies *(P* = 0.73). The patients with hypertension in the early invasive strategy were 17.9%, and less than 18.8% in the delayed invasive strategy (*P* = 0.96). The history of previous myocardial infarction, previous PCI and previous CABG accounted for 19.3%, 14.0% and 7.4% respectively. There was no significant difference on previous cardiovascular history (0.07≤*P≤*0.67). The relevant history of cardiovascular disease was not supplied in Sciahbasi and Tekin trial [13,14]. No significant difference in ages, hypertension, hyperlipemia, diabetes, and history of cardiovascular disease were found by one-way analysis of variance between early and delayed invasive strategies (one-way ANOVA) (*P*>0.05). Average follow up period was 18.5 months, ranging from 3 months in Tekin to 60 months in OPTIMA [14,15].

The procedural characteristics for patient included study are shown in Table 3. The average time of randomization in in the early invasive strategy was significantly lower than that of the delayed invasive strategy [3.8h, (IQR 1.475–11.75) vs 24.5 h, (IQR 24–43.75)]. Most of patients in each study underwent coronary angiography. The PCI, CABG were definitively included into the both invasive strategies. Only patients undergoing PCI were included in the OPTIMA trial. The time to intervene for the early invasive strategy varied from 0.5 h to 24 h, while that of the delayed invasive strategy ranged from 18.3 h to 54.9 h. The proportion for PCI in the early invasive strategy was from 59.6% to 100%, while that of the delayed invasive strategy was from 55.1% to 99%. A loading dose of 300 mg clopidogrel was taken in the three trials [13,16,17], the loading dose for the remaining studies was 600 mg [10,16,18].

**Table 3:** Procedural characteristics and medicinal therapy for patient included study.

	ELISA3		LIPSIA		OPTIMA		Sciahbasi		Tekin		TIMACS	
	Early	Delayed	Early	Delayed	Early	Delayed	Early	Delayed	Early	Delayed	Early	Delayed
**MTA(h)**	2.6	54.9	1.1	18.3	0.5	25	5	24	<24	24–72	14	50
**NCV 1 (%)**	27.7	26.2	32	53/198	41	54	63	63	13	17.7	31.6	31.1
**NCV2 (%)**	29.2	35.5	30	64/198	45	32	33	30	58.1	25.8	24.5	23.4
**NCV3 (%)**	33	26.6	30	54/198	95	93	4	7	58.1	56.5	17.1	15.8
**LAD (%)**	37.7	34.7	32	66/198	NP	NP	33	33	55.1	54.8	NP	NP
**RCA (%)**	16.5	25.3	24	38/198	NP	NP	26	22	15.9	19.4	NP	NP
**LCX (%)**	22.2	22.2	32	46/198	NP	NP	41	45	29	25.8	NP	NP
**LM (%)**	0.4	3.1	2	6/198	NP	NP	NP	NP	0	0	10	9.5
**Graft (%)**	9.7	6.7	1	2/198	NP	NP	NP	NP	NP	NP	NP	NP
**PCI (%)**	66.7	61.9	76	71	100	99	78	59	NP	NP	59.6	55.1
**CABG (%)**	23.2	25.7	8	13	NP	NP	NP	NP	NP	NP	14.8	13.6
***β*-Blocker (%)**	81.2	83.1	99	99	96	96	NP	NP	NP	NP	86.8	86.9
**ACEI/ARB (%)**	55.5	49.4	99	99	49	48	NP	NP	NP	NP	74.2	73.6
**Statin (%)**	85.3	81.9	99	97	97	96	NP	NP	NP	NP	85.1	84.3
**Aspirin (%)**	78.8	75.1	100	100	100	97	NP	NP	NP	NP	98	98.1

**Abbreviations**: MTA: median time angiography; NCV: number of culprit vessel; LAD: left anterior descending artery; RCA: right coronary artery; LCX: left circumflex artery; LM: left main; PCI: percutaneous coronary intervention; CABG: coronary artery bypass grafting; β-Blocker: beta receptor blocker; ACEI: angiotensin-converting enzyme inhibitors; ARB: angiotensin receptor blocker.

### Quality assessment and risk of bias

The quality of most randomized controlled trials was higher according to the Cochrane quality assessment criteria (S1 Fig). The weight of studies was not affected by the quality of studies that could be used as an indicator of validity.

### Clinical outcomes

**Fig 2:**
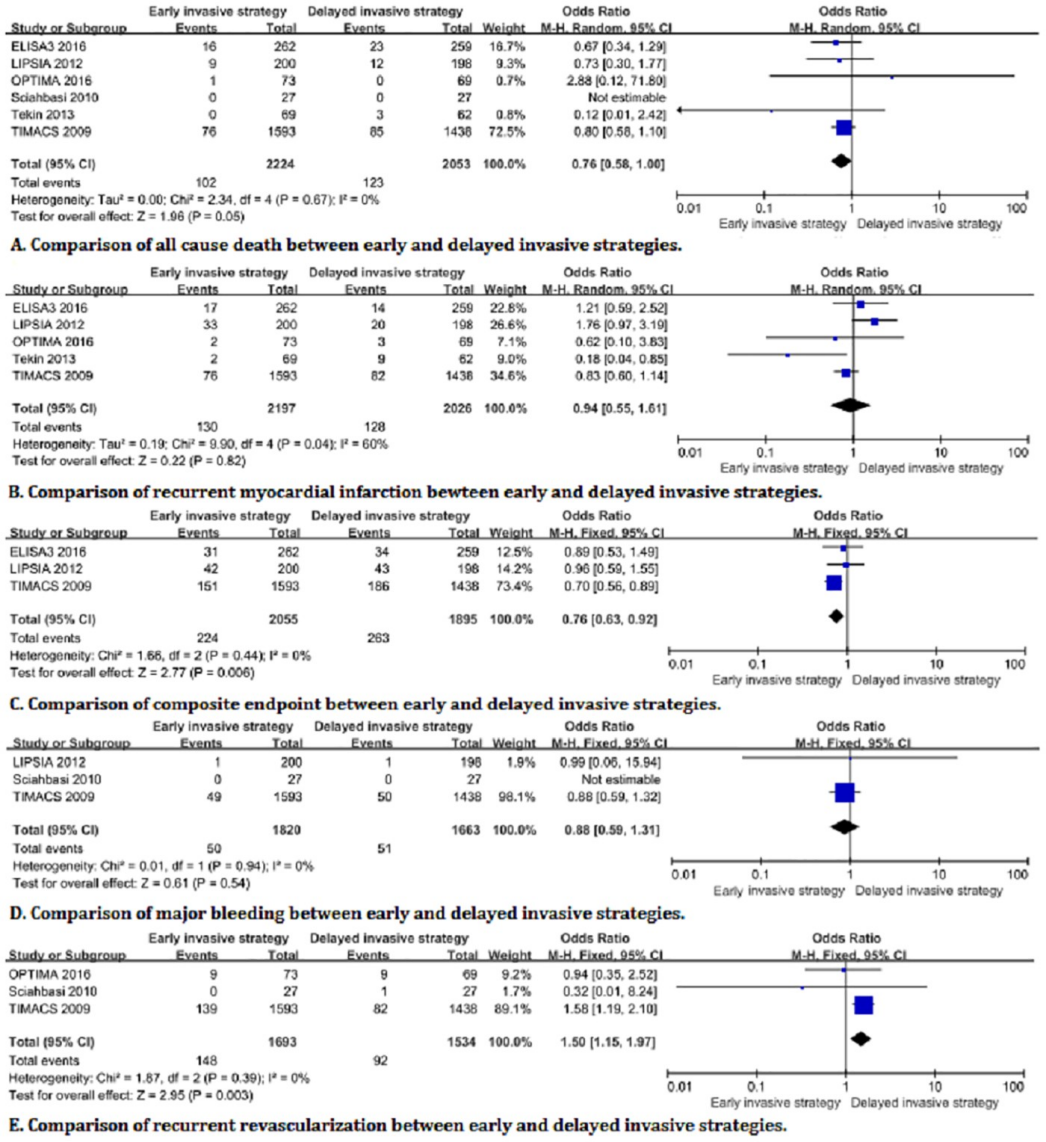
Forest plots of related endpoints between early and delayed invasive strategies.

**Fig 3:**
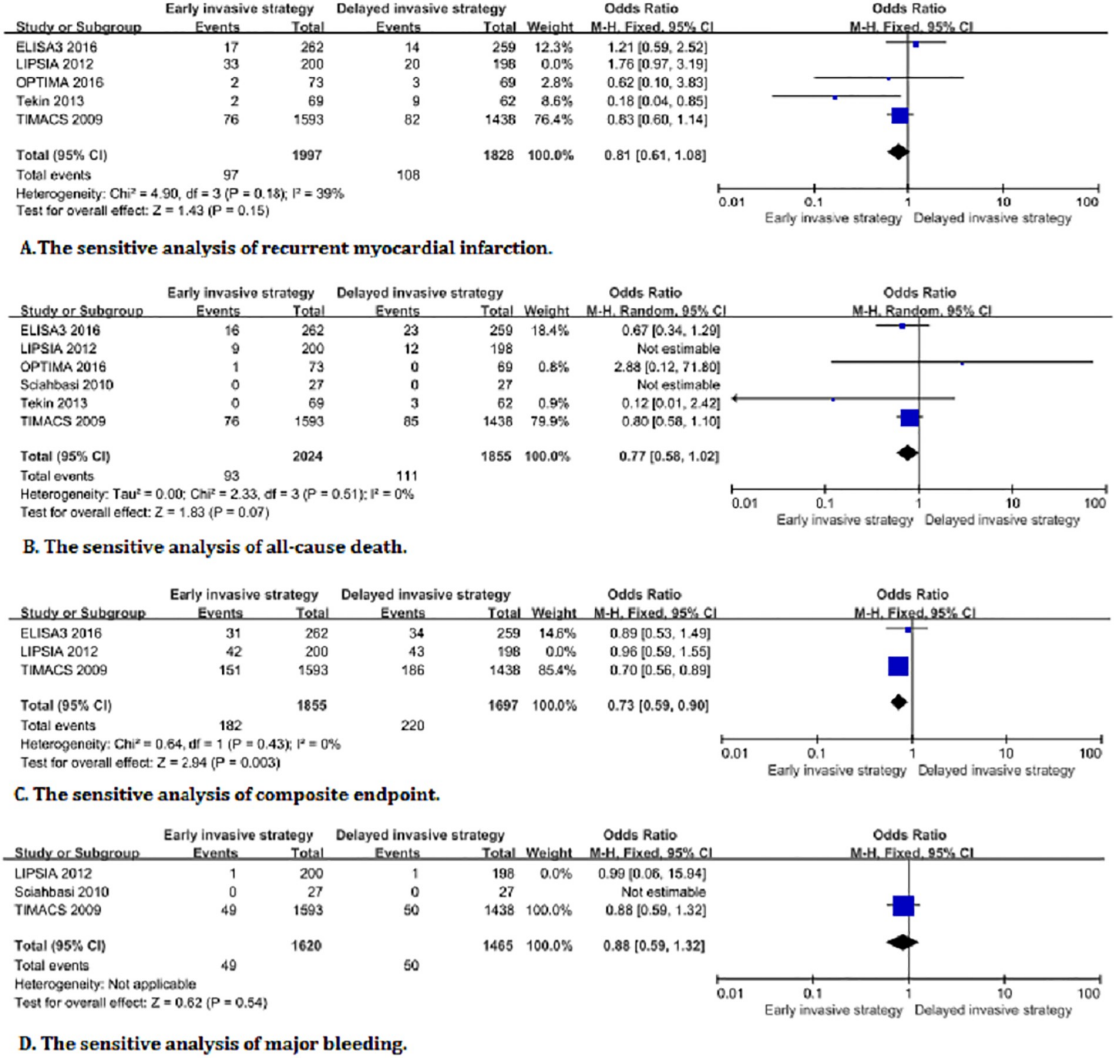
The sensitive analysis excluding the LIPSIA trial on related endpoints between early and delayed invasive strategies.

The primary endpoints analysis showed that the all-cause death accounted for 4.6% (102/2224) of the early invasive strategy and 6% (123/2053) of the delayed invasive strategy (Fig 2A). There was a significant difference in all-cause death between both invasive strategies (OR:0.76, 95%CI:0.58–1.00, *I*^*2*^ = 0%, *P* = 0.05). Meanwhile, the recurrent myocardial infarction accounted for 6% (130/2179) of the early invasive strategy, and 6.3% (128/2026) of the delayed invasive strategy. However, substantial heterogeneity is observed among studies for the recurrent myocardial infarction (OR: 0.94, 95% CI:0.55–1.61, *P* = 0.82, *I*^*2*^ = 60%) (Fig 2B). A significant heterogeneity was shown among studies for the recurrent myocardial infarction, but the pooled results were still not statistically significance when the LIPSIA trial was removed from the forest plots of the recurrent myocardial infarction by sensitivity analysis. The result of heterogeneity was significantly decreased to 39% (OR: 0.81, 95% CI:0.61–1.08, *P* = 0.15, *I*^*2*^ = 39%), but the pooled result was not affected by the significant heterogeneity (Fig 3A).

**Fig 4:**
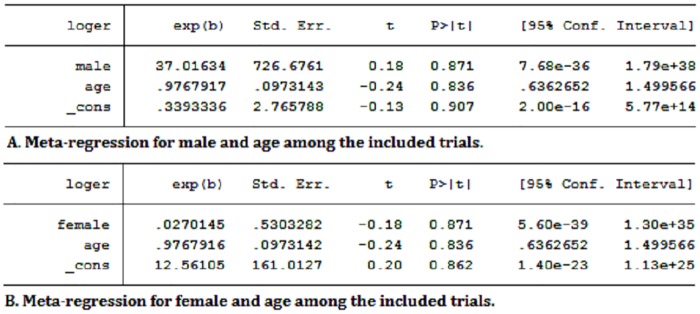
Meta-regression for gender and age.

The results of the secondary endpoint analysis demonstrated that the incidence of the composite endpoint was 10.9% (224/2055) for the early invasive strategy and 13.9% (263/1895) for the delayed invasive strategy in the three trials [10,17,19]. The composite endpoint of the former is slightly lower than that of the latter (OR: 0.76, 95%CI:0.63–0.92; *P* = 0.006, *I*^*2*^ = 0%) (Fig 2C). There is no significant difference in the incidence of major bleeding in patients with NSTE-ACS between early and delayed invasive strategies (2.7% (50/1,820) vs 3.1% (51/1,663); OR: 0.88, 95%CI: 0.59–1.31; *P* = 0.54, *I*^2^ = 0%) (Fig 2D). The recurrent revascularization is significantly higher in the early invasive strategy than that of the delayed invasive strategy (8.7% (148/1,693) vs 0.6% (92/1,534); OR:1.50, 95%CI:1.15–1.97; *P* = 0.003, *I*^*2*^ = 0%) (Fig 2E). Because the immediate and early invasive strategies in the LIPSIA trial were considered as the early and delayed invasive strategies in other trials respectively, the overlapping definitions of invasive strategy might potentially lead to bias in results. Therefore, sensitivity analysis was performed for the relevant endpoints, which showed no significant difference in other endpoints compared with removing the forest plot before the LIPSIA trial except for all-cause death. There is no statistical difference among the pooed results after removing the LIPSIA trial from the forest plot of the all-cause death group (Fig 3B–3D). The funnel plot of the meta-analysis was not performed due to less than 10 trials in the included trials. Age and gender were the risk factors for coronary artery disease, which might have potential impact on each trial. However, the meta-regression analysis is performed and showed no significant heterogeneity (Fig 4).

## Discussion

The principal results of the present meta-analysis are: (1) there was a significant reduction in all-cause death in patients with NSTE-ACS in the early invasive strategy. (2) the incidence of recurrent myocardial infarction in the primary and secondary endpoints excluding recurrent revascularization in the early invasive strategy patients with those were not less than those of the delayed invasive strategy during short-medium follow up. However, the recurrent revascularization was significantly higher in the early invasive strategy.

The ESC guideline recommended the invasive coronary strategy for patients with NSTE-ACS according to different risk stratification of patients with those (Class I; Level of Evidence A) [20]. Meanwhile, the subgroup analysis of TIMACS study showed that the early invasive strategy (<24 h) was performed for the high-risk patients with NSTE-ACS [21], which could improve the composite endpoint of myocardial ischemia and death. There was a contradiction with the previous publish results of meta-analysis [19,22,23]. Significant differences regarding death were not detected in the previous studies. However, this meta-analysis found that the early invasive strategy had reduced significantly the all-cause death comparing with the delayed invasive strategy during at least three months of follow up. Because the *P* value was equal to 0.05 in our study, which was located at the marginal value with statistical significance. The marginal *P* value might be associated with few participants in our study and inconsistent results attributing to the shorter follow-up duration of previous studies. For example, death toll in a previous study by Sciahbasi et al was not detected due to the low participants and short follow up [13]. In the SWEDEHEART study, an early invasive treatment reduced all-cause mortality compared with delayed invasive treatment, which was mainly targeted at those with severe heart failure, refractory angina, fatal arrhythmia. The conclusions of the TIMACS trial was confirmed in the SWEDEHEART study [24], but the conclusions of the KAMIR-NIH study were reversed [25]. In the TIMACS trial, the early PCI could obtain benefits on death from the high risk patients, such as the elderly, GRACE scores>140 [19]. The GRACE score based on risk factors in this study were only used for the assessment of deaths within six months of follow-up [26,27], while the results of death predictions for follow-up over six months were inconclusive. When the LIPSIA trial was removed from the forest plot of the all-cause death group, previous statistic difference was not shown in the pooled result. The reason for no statistical difference in sensitivity analysis might be earlier time of invasive strategy in the LIPSIA than that of the other trials. Since the numbers of most deaths in the meta-analysis originated from the TIMACS trial, which concluded that the early and delayed invasive strategies did not differ from death within six months of follow-up [19]. The weight of the study in the meta-analysis was significantly higher than that of other included studies, and a higher impact was showed on the synthesis results of the meta-analysis. The conclusion of obtaining all-cause death benefit with the early invasive strategy in the meta-analysis should be cautiously explained due to the results of the meta-analysis synthesis were opposite to that of the TIMACS trial. Therefore, it is necessary to implement large sample size and long-term follow up randomized controlled trials to be confirmed further.

There was no significant difference in recurrent myocardial infarction between the two strategies, but a heterogeneity was illustrated in the result of recurrent myocardial infarction. The reason for heterogeneity change was attributed to the selective bias. For example, three strategies were included in the LIPSIA-NSTEMI trial [10]: immediate group, early group and selective group, but pooled results were only found in the immediate and early groups. The potential benefits of the early invasive strategy for the recurrent myocardial infarction were covered by the different definition in the included studies [6]. Because the definition of myocardial infarction was various in each study, previous studies included spontaneous and perioperative myocardial infarction. The impact of two invasive strategies on spontaneous myocardial infarction was assessed, but the perioperative myocardial infarction could not be excluded from the included trials. In addition, it was difficult to evaluate the recurrent myocardial infarction in clinical trials when myocardial biomarkers fail to return normal or were at a declining phase. Therefore, increased number of myocardial infarction and ischemic complications had led to the insignificant outcome [28]. For this reason, the recurrent myocardial infarction should also be further studied in patients with NSTE-ACS according to the universal definition of myocardial infarction.

The early invasive strategy was also supported by the composite endpoints (all-cause death, myocardial infraction, myocardial ischemia) in the meta-analysis. However, the target population of very high-risk patients could not be included in the trials, which might affect the secondary endpoints. Meanwhile, the results of previous trials were mainly the results of short-term follow up. In a word, the early invasive strategy had significant benefit on the composite endpoints during short-medium follow up.

There was no statistical difference in major bleeding between early and delayed invasive strategies, and this meta-analysis also showed no heterogeneity of major bleeding. However, the meta-analysis by Yanda Li et al showed that the incidence of major bleeding was lower among early adopters of invasive strategy [29]. Therefore, it is suggested that the early invasive strategy should be applied to prevent major bleeding for patients with high risk of bleeding, such as the elderly, hepatic and renal insufficiency.

This study showed that the number of recurrent revascularization could be reduced by the delayed invasive strategy in short-medium term follow up, which was consistent with previous study by Rajpurohit [22]. The TIMACS trial reported recurrent ischemia and repeat revascularization separately [19], only ischemia events required additional intervention considering as episodes of recurrent ischemia [30]. The rate of recurrent revascularization in patients underwent early PCI was higher in the ACUITY trial, which could be attributed to the high formation of thrombus due to a high rate of abrupt closure [23], and repeat revascularization was ascribed to extensive vascular disease requiring early intervention or severe systemic inflammation in patients with NSTE-ACS from the PURSUIT trial [31]. Meanwhile, it is not clear why the delayed invasive strategy could decrease the frequency of recurrent revascularization.

Given the potential impact of age and gender in each trial, a meta-regression analysis was performed and showed no significant heterogeneities, which might be related to younger age of the patients, male dominance and different surgical styles in the included trials.

## Limitations

This study was limited by certain factors. Firstly, the number of RCTs included was small with only six. The included population of this study was smaller comparing to the previous meta-analysis. Secondly, there was the heterogeneity in the aspect of recurrent myocardial infarction, a random effects model might reduce the impact of the heterogeneity on the result. Thirdly, some studies less than three months follow-up period did not include to assess the short-medium term outcome of the early and delayed invasive strategies. Fourthly, the time overlap of the early and delayed invasive strategies in the included trials could easily result in the bias of results. Fifthly, other endpoints related to invasive strategies (e.g., left ventricular ejection fraction, antithrombotic treatments, the style of procedures) were not assessed due to the different purpose of the meta-analysis. Sixthly, since most of the weight of each endpoint were based on the TIMACS trial, the results were mainly related to the TIMACS trial. Seventhly, the present study is a meta-analysis of aggregate data rather than an individual participant data meta-analysis without decreasing the limitations in the meta-analysis. Lastly, the incomplete revascularization of coronary artery was adopted in the included trials, which might lead to differences in the relevant endpoints.

## Conclusion

The systematic review and meta-analysis demonstrated that the early invasive strategy had a beneficial trend on all-cause death and significantly reduced the composite endpoint in patients with NSTE-ACS, but increased the rate of revascularization. These data could provide a solution for patients with those.

## Supporting information

S1 Fig The quality assessment of the included trials. (TIF)

S1 Table PRISMA checklist. (DOC)
