# Supplementary figures and images for: Comparison of early and delayed invasive strategies in short-medium term among patients with non-ST segment elevation acute coronary syndrome: A systematic review and meta-analysis
Source: PLoS One. 2019 Aug 12;14(8):e0220847. doi: 10.1371/journal.pone.0220847 (PMC6690510; doi:10.1371/journal.pone.0220847)

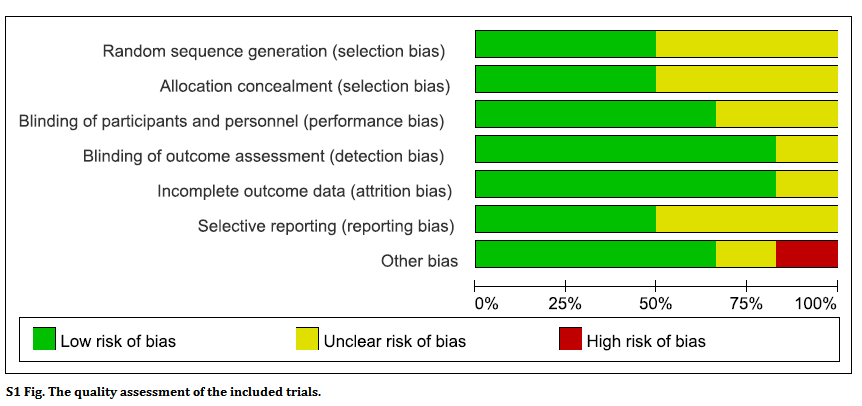

Supplement: S1 Fig — (TIF) [file pone.0220847.s001.tif]
